# Investigating the Immune Effects of Radiotherapy in Non-Small Cell Lung Cancer—Results of the PD-RAD Study

**DOI:** 10.32604/or.2025.072053

**Published:** 2026-02-24

**Authors:** Shuhui Cheng, Tiana Kordbacheh, Antonia Banyard, Anshuman Chaturvedi, Diego Sanchez Martinez, Crispin T. Hiley, Maggie Harris, Clara Chan, Corinne Faivre-Finn, Timothy M. Illidge, Eleanor J. Cheadle

**Affiliations:** 1 Targeted Therapy Group, Division of Cancer Sciences, School of Medical Sciences, Faculty of Biology, Medicine and Health, University of Manchester, Manchester Academic Health Science Centre, Manchester, M20 4BX, UK; 2 Cancer Research UK Manchester Institute, The University of Manchester, Manchester, M20 4BX, UK; 3 The Christie NHS Foundation Trust, Manchester, M20 4BX, UK; 4 University College London Hospitals NHS Foundation Trust, London, WC1E 6AG, UK; 5 Cancer Research UK Lung Cancer Centre of Excellence, University College London Cancer Institute, London, WC1E 6BT, UK

**Keywords:** Non-small cell lung cancer (NSCLC), radiotherapy, tumor microenvironment, biomarker, programmed death-ligand 1 (PD-L1), classical monocytes

## Abstract

**Objectives:**

The PACIFIC trial established the benefit of durvalumab following chemo-radiotherapy for stage III non-small cell lung cancer (NSCLC). However, the concurrent use of radiotherapy (RT) and durvalumab (PACIFIC-2 trial) showed no additional advantage. The PD-RAD study was set up to understand the immunological effects of RT on the tumor microenvironment (TME) to aid in optimizing sequencing of combination therapies.

**Methods:**

The PD-RAD trial (ClinicalTrials.gov identifier: NCT03258788) aimed to enroll thirty NSCLC patients receiving radical-intent RT. Tumor biopsies and blood samples were collected pre-RT and at week 2 during RT and analyzed using multiplex immunohistochemistry (mIHC) and high-dimensional mass cytometry (CyTOF), respectively.

**Results:**

Paired biopsies were collected from only three patients (Pts 1, 3 & 4) and blood from four patients (Pts 1–4) before the study was closed early during the COVID-19 pandemic. Programmed Death-Ligand 1 (PD-L1) expression in the TME was raised in Patient 1, who responded well to treatment, and unaltered in two patients with progressive disease. CyTOF analysis revealed elevated circulating classical monocytes, highest in the patient with a good response.

**Conclusions:**

This study underscores the challenges of integrating advanced immune monitoring during RT delivery and did not meet its primary endpoint. The hypothesis-generating findings highlight PD-L1^+^ macrophages in the TME and classical monocytes in the blood as potential immune biomarkers of RT response, but larger studies are needed to validate these observations and characterize the immune changes following curative-intent RT in patients with NSCLC.

## 1 Introduction

RT plays an important role in the management of early and locally advanced NSCLC [1,2]. In recent years, substantial developments have been made in NSCLC, including disease concepts, drug development, and treatment options [1,2]. Anti-programmed death-ligand-1 (PD-L1) immune checkpoint inhibitors (ICIs) are now used as standard-of-care in both advanced and locally advanced disease settings. The pivotal phase 3 PACIFIC trial led to standard-of-care addition of consolidative durvalumab (anti-PD-L1) for patients with inoperable stage III NSCLC without disease progression following 2 or more cycles of consolidative chemo-radiotherapy (CCRT), based on superior progression-free survival (PFS) and overall survival (OS) [3]. Updated 5-year OS was 42.9% vs. 33.4% (placebo), respectively.

NSCLC is a highly heterogeneous disease, and the dynamics between tumor and immune cells within the tumor microenvironment (TME) can determine disease progression and response to therapy [4–7]. PD-L1 and tumor mutation burden (TMB) have been demonstrated as prognostic biomarkers for favorable response to ICIs in both early and advanced NSCLC [8,9], but no such biomarkers exist for response to (C)RT alone. Whilst tumor cell PD-L1 expression has been shown to be increased during RT in preclinical models, driven by IFNγ producing CD8^+^ T-cells in a process termed adaptive resistance [10], and differentially expressed clinically in patients receiving CRT [11,12], understanding the dynamic regulation of PD-L1 during chemoradiotherapy is crucial for optimizing the timing of ICI administration. A post hoc analysis of the PACIFIC study showed that patients with PD-L1 >1% derived the most benefit from durvalumab [13]. Additionally, while PD-L1 expression and chronological immune alterations have been extensively studied in the context of response to immunotherapy, dynamics during chemo-radiotherapy remain relatively understudied. Furthermore, whilst liquid biopsies have mainly focused on circulating tumor-free DNA for lung cancer [14], the potential role of systemic immune biomarkers in NSCLC remains unclear [15]. Predictive tumor and peripheral circulating biomarkers associated with RT are therefore needed to enhance our understanding and guide the shift towards more personalized treatment strategies for NSCLC.

***Aims***

The PD-RAD study was therefore set up to investigate whether PD-L1 expression levels increase during radiotherapy in early/locally-advanced NSCLC patients receiving standard of care (chemo)radiotherapy and to identify potential systemic immune biomarkers of response.

## 2 Materials and Methods

The PD-RAD study was conducted in accordance with the Declaration of Helsinki and approved by the NHS North West-Liverpool East Research Ethics Committee (IRAS study ID: 180593 on 21 November 2017, ClinicalTrials.gov identifier: NCT03258788, Sponsor: The Christie NHS Foundation Trust). Translational analyses were approved by the Yorkshire & The Humber—Leeds West Research Ethics committee (IRAS study ID: 297993 on 22 November 2021, Sponsor: The University of Manchester). The PD-RAD study (A translational study investigating PD-L1 expression after RADiotherapy for NSCLC) was designed to prospectively investigate chronological immune microenvironment dynamics at baseline (pre-RT), during RT (RT alone or sequential RT), and post RT in patients with inoperable NSCLC, and determine the feasibility of obtaining an additional lung biopsy during the second week of RT to compare with baseline archival tissue.

Inclusion criteria included patients aged ≥18 years with histologically confirmed inoperable NSCLC deemed suitable for RT (palliative or radical) or sequential CRT, baseline biopsy considered suitable for PD-L1 analysis (>100 tumor cells), tumor deemed accessible for a repeat core biopsy, and ECOG Performance status (PS) 0–2. Exclusion criteria included participants deemed unsuitable for repeat biopsies, advanced stage IV NSCLC with known EGFR mutation or ALK rearrangement (thus suitable for tyrosine kinase inhibitors), intercurrent or past history of hepatitis B, C or human immunodeficiency virus (if known), participants who have received more than 1 line of chemotherapy prior to radiotherapy or any line of immunotherapy and patients with other active malignancies or treated malignancy within the last 12 months. The primary endpoint was to attain paired archival and repeat on-treatment biopsies for PD-L1 assessment in 21 of 30 evaluable patients.

**Figure 1:**
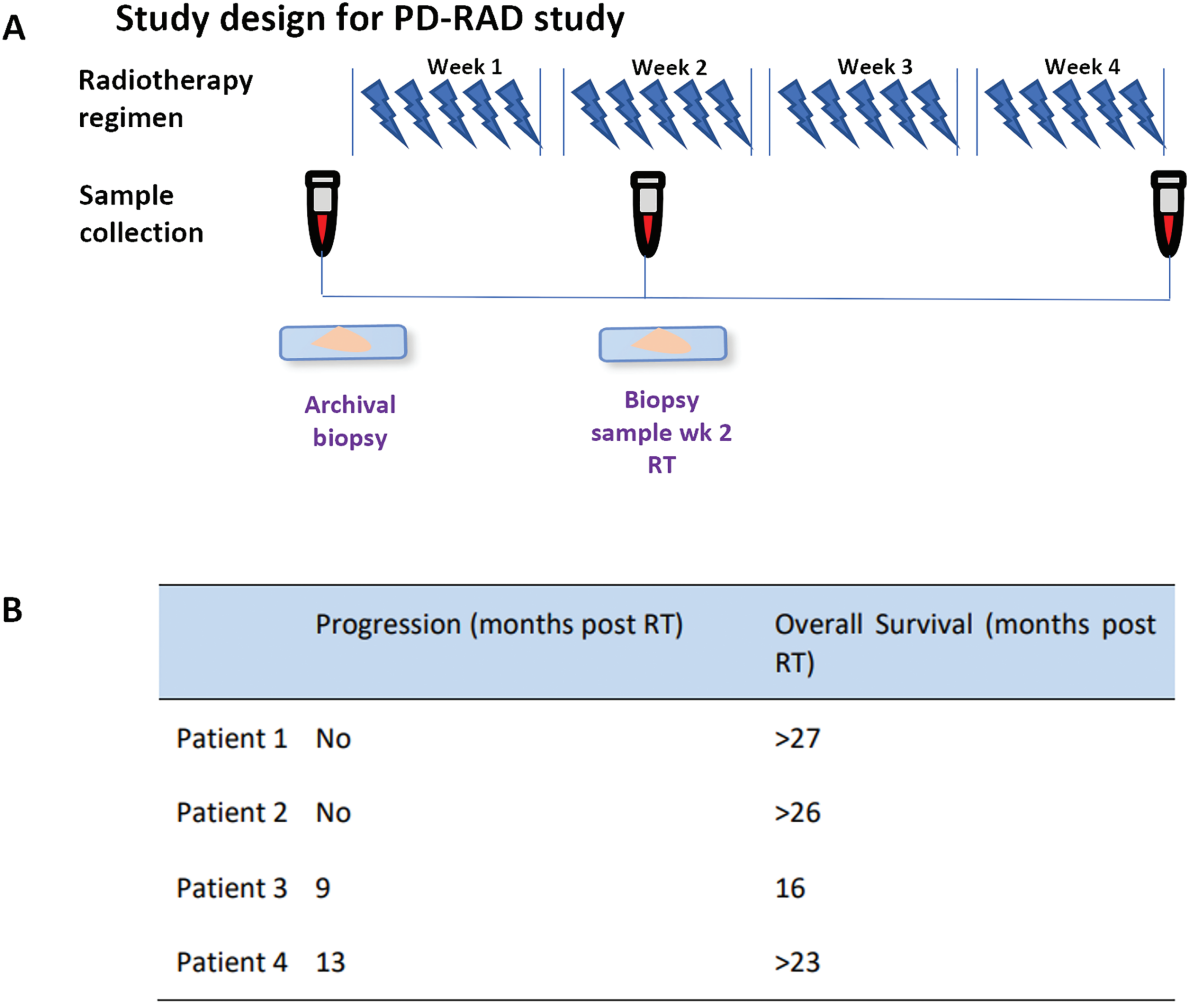
(**A**) Study design for the PD-RAD study detailing radiotherapy regime and sample collection. (**B**) Patient clinical outcomes tabulated as progression (no progression or month of progression post recruitment to study) and overall survival (months post RT). > represents patient still alive at final follow up (June 2021). Abb: RT, Radiotherapy

All patients underwent informed consent, with additional consent provided to undergo the additional paired on-treatment biopsy during week 2 of RT. Pre-RT archival biopsies were retrieved from the relevant NHS hospital pathology department. Blood samples were collected at baseline, repeat biopsy (week 2), and upon completion of RT (Fig. 1A). For baseline biopsies to be adequate for PD-L1 expression analysis, tumor tissue had to be formalin fixed for >12 h and ≤24 h, have tumor tissue and morphology confirmed by H&E staining, and contain sufficient tumor cells (>100), as confirmed by central histopathologist review. Peripheral blood mononuclear cells (PBMCs) were isolated and cryo-frozen for later thawing and processing using cytometry by time of flight (CyTOF). Ultivue multiplex immunohistochemistry (mIHC) was utilized to identify immune cell phenotypes in paired diagnostic and during-RT biopsies.

### 2.1 H&E Staining of Tumor Biopsies

Formalin-fixed paraffin-embedded (FFPE) specimens were cut as 4 µm sections on glass slides. Sections were dewaxed by soaking in xylene (Gentamedical, XYL050, York, UK) for 3 × 8 min, followed by rehydration by passing through a series of decreasing alcohol concentrations (100% × 3, 90%, 70%, Gentamedical, 199050) for 1 min through to water. Sections were then stained in Gills 2 hematoxylin (3 min) (Epredia, Fisher Scientific, 10096648, Loughborough, UK), rinsed in water, washed in mild acid (1% acetic acid, Avantor, 1.00063.1011, Lutterworth, UK), dipped in bluing reagent (Scotts tap water, Leica Biosystems, 3802901E, Milton Keynes, UK) for 1 min, rinsed in water, dipped in Eosin (Epredia, Fisher Scientific, 6766008) for 45 s and then rinsed in water. Slides were then dehydrated by passing through a series of increasing concentrations of alcohol (70%, 90%, 100% × 3) followed by xylene (3 × 8 min) before coverslipping using a Pertex mounting medium (CellPath, SEA-0100-00A, Newtown, UK).

### 2.2 Ultivue^***®***^ Immunohistochemical Analysis of Tumor Biopsies

FFPE specimens were sectioned into 4 µm slides. DNA-barcoded multiplexed immunofluorescence (mIF) technology was utilized via the FixVUE Immuno-8 Kit (Ultivue Inc., ULT30801, Cambridge, MA, USA); markers included Cluster of Differentiation 3 (CD3), PD-1, PD-L1, CD68, CD8, CD4, FoxP3, and pan-CK/SOX10 cocktail used at a 1 in 100 dilution. The Leica Biosystems BOND RX autostainer (Leica Biosystems, 21.2821, Sheffield, UK) was used to perform H&E and mIF staining.

FFPE slides were deparaffinized at 60°C–65°C for 30 min. Slides were stained following the Leica BOND RX and Ultivue Immuno-8 kit manufacturer’s instructions, which are described briefly below.

Every slide was first labelled with a combination of all 8 of the conjugated antibodies (60 min at ambient temperature), after which assay sensitivity was increased by simultaneously amplifying the DNA barcodes of each target (90 min). Two sequential rounds of detection were then performed by binding and labelling four immune marker targets with fluorescent probes and complementary DNA barcodes (25 min); after each round, a fluorescent image was captured, the cover slip was removed (soaked in PBS), the fluorescent signal was removed, and the next round of probes was applied with ensuing imaging. Round 1 included DAPI, CD8, PD1, PDL1, CD68 and round 2 included CD3, CD4, FOXP3, PanCK. No quenching, bleaching, or other techniques to reduce signal between rounds were required. Stained slides were cover-slipped (Fisher brand Cover Glass 22 40 mm, #1.5) and mounted with Prolong Gold Anti-Fade mountant (Thermo Fisher Scientific, cat# P36965, Loughborough, UK) prior to each imaging session. The following Immuno8 images marker-fluorophore pairings were used: FITC (CD8 Round 1 (R1), CD3 Round 2 (R2)), TRITC (PD-1 R1, CD4 R2), Cy5 (PD-L1 R1, FoxP3 R2), and Cy7 (CD68 R1, panCK/Sox10 R2).

An Olympus VS120 high-resolution microscope (Olympus Corporation, VS120-L100-W-12, Tokyo, Japan) was used to scan rounds of stained slides (magnification of 20), generating digital images in VSI format. HALO v3.1.1076.429 software (Indica Labs, New Mexico, United States) was used to analyze images by applying machine learning algorithms; imported images were fused with the DAPI channel to serve as a nuclear counterstain, with applied focusing and alignment using 9 channels (nuclear stain and 8 immune markers) to generate composite images. Cell (nuclear) and cytoplasmic segmentation were applied and quality-adjusted on random groups of cells to reduce the frequency of over- and under-segmentations.

Individual biomarker signal thresholds were defined by visual assessment of independent images. Histopathologist-marked tumor and stromal regions of interest were mapped to composite imaged slides and a Random Forest module tissue/background classifier was applied. Marker positivity was determined by setting signal thresholds using the HighPlex FL v3.2.1 module. Cellular phenotypes were determined by using a combination of positive and negative signal criteria for each marker. Data analysis and visualization were performed with GraphPad Prism version 9 (GraphPad, Prism V9, San Diego, CA, USA) (n = 1).

### 2.3 Immuno-Phenotyping of PBMC by CyTOF

PBMC aliquots were thawed and reconstituted in 10 mL of serum-free (sf) RPMI (Sigma Aldrich, R0883, Poole, UK), centrifuged at 400× *g* for 5 min at room temperature, resuspended in 1 mL sf RPMI plus 5 mM Mg^2+^ (Sigma Aldrich, M1028) with 0.2 mg/mL DNase I (Sigma Aldrich, D4263), and incubated at 37°C for 10 min. After cell counting, cells were resuspended in Maxpar^®^ Cell Staining Buffer (CSB, Standard Biotools™, Catalogue Number 201068, Cambridge, UK) at 12 × 10^6^/mL, with 3 × 10^6^ cells utilized for CyTOF analysis.

Standardized (3 × 10^6^) PBMC aliquots were incubated in 1 µm ^198^Pt Cisplatin (Standard Biotools, 201198) for ten seconds, washed in CSB, and incubated in 20 µL Heparin solution (Sigma Aldrich, H3149) with 2 µL Fc block (Trustain FcX block, Biolegend, 422301, London, UK), at room temperature for 5 min. Samples were incubated in 50 µL of extracellular antibody mastermix (Table S1) for 45 min on ice. After washing in CSB, PBMC were fixed and incubated in 1 mL FoxP3 Fix Perm buffer (eBioscience, Thermo Fisher Scientific, 00-5523-00) for 30 min at room temperature. Cells were washed again with CSB and re-suspended in 1 mL 10% DMSO-containing CSB solution. These samples were stored at −20°C for future intracellular labelling and barcoding.

Frozen extracellular-labelled PBMC samples were thawed at room temperature, resuspended in 2 mL of CSB, centrifuged at 1000× *g* for 6 min at room temperature, and washed with PBS. Washed cells were re-suspended in 1 mL of ice-cold PBS containing the Standard Biotools cell ID 20-Plex Pd barcoding labels (Standard Biotools, 201060). After incubation at room temperature for 15 min, barcoding was quenched with 3 mL of CSB followed by two CSB washes, and resuspension in 4 mL of FoxP3 perm buffer in a single tube. After centrifugation at 1000× *g*, cells (6 min, room temperature) were re-suspended in 20 µL of heparin for every 3 × 10^6^ cells, and 1 µL Fc block was added for 5 min at room temperature.

For every 3 × 10^6^ cells, PBMC were incubated in 50 µL intracellular antibody mastermix (Table S1) for 45 min in the dark at room temperature. Cells were washed in CSB and fixed in 1 mL of 4% paraformaldehyde (PFA) in PBS (fixation buffer, Biolegend, 420801) per 3 × 10^6^ cells, for a minimum of 18 h. Finally, per 3 × 10^6^ cells, 1 µL of iridium solution (10 mg/mL in 0.1 M NaOH in Standard Biotools Maxpar water, 201192) was added and incubated at room temperature for 1 h. Labelled and fixed PBMC were washed and centrifuged at 1000× *g* for 6 min at room temperature three times in PBS followed by water, re-suspended at 1 × 10^6^ cells/mL in 15% EQ beads with water (Standard Biotools, 201245), and filtered twice through 70 µm filcons (Becton Dickinson, 340605 Oxford, UK).

PBMC acquisition via the Fluidigm Helios Mass Cytometer’s super sampler (Standard Biotools, Helios) was set at 30 µL per minute with 300–500 events per second. FlowJo software (v10.7, Becton Dickinson, Ashland, OR, USA) was utilized to eliminate acquisition artefacts, applying Gaussian gating, exclusion of EQ calibration beads, selecting single cells, and gating for live cells (negative for 198Pb Cisplatin) and CD45^+^ (141Pr) cells.

Cytobank software v10.3.4 (Beckman Coulter, MRC server, Amersham, UK) was used for data visualization. FCS files were cleaned using PeacoQC and run through a tSNE-CUDA dimensionality reduction. Unsupervised clustering was performed with the FlowSOM algorithm using equal numbers of events (74,750) and 20 metaclusters and a random seed number. Cell population identification was carried out manually based on median channel expression within each cluster using raw data and heatmaps expressing standardised z-scores. The percentage of cells within each cluster was calculated for each sample.

### 2.4 Statistical Testing

Statistical analysis assessing potential correlations between immune cell levels and progression-free survival was performed using simple linear regression analyses, where appropriate, using GraphPad Prism v9.

## 3 Results

### 3.1 The PD-RAD Study

PD-RAD study recruitment commenced in early 2019 and was closed early in March 2020 secondary to the COVID-19 pandemic due to concerns over the vulnerability of this patient group to COVID-19. Of 19 patients who expressed initial interest in the study and received a patient information sheet, 13 patients declined participation; 9 patients specifically declined due to the need for repeat biopsy, and the remaining patients gave no reason. A total of 6 patients were enrolled, of whom 5 patients proceeded to RT. Paired biopsies (archival and week 2-RT) were feasible in only 3 patients (patient 2 declined a second biopsy as a fresh baseline biopsy was taken as part of the study and patient 5 was deemed unfit at point of procedure) and serial blood samples (baseline, during RT, and end of RT) were achieved in 4 patients. Consequently, the study was deemed not to meet the primary endpoint of achieving paired biopsies in 21 of 30 evaluable participants, highlighting the challenges of obtaining on-treatment biopsies in this challenging patient cohort.

Fig. 1A demonstrates sample collection timing, and clinical outcome data are shown in Fig. 1B. Of the 3 patients with paired biopsies, only Patient 1 showed no progression within the two-year follow-up period and was consequently termed a good responder. Whilst CT scans showed a partial response, five years following treatment the patient still experienced stable disease with no progression or further treatment required.

### 3.2 TME Immuno-Phenotyping Before and During RT

**Figure 2:**
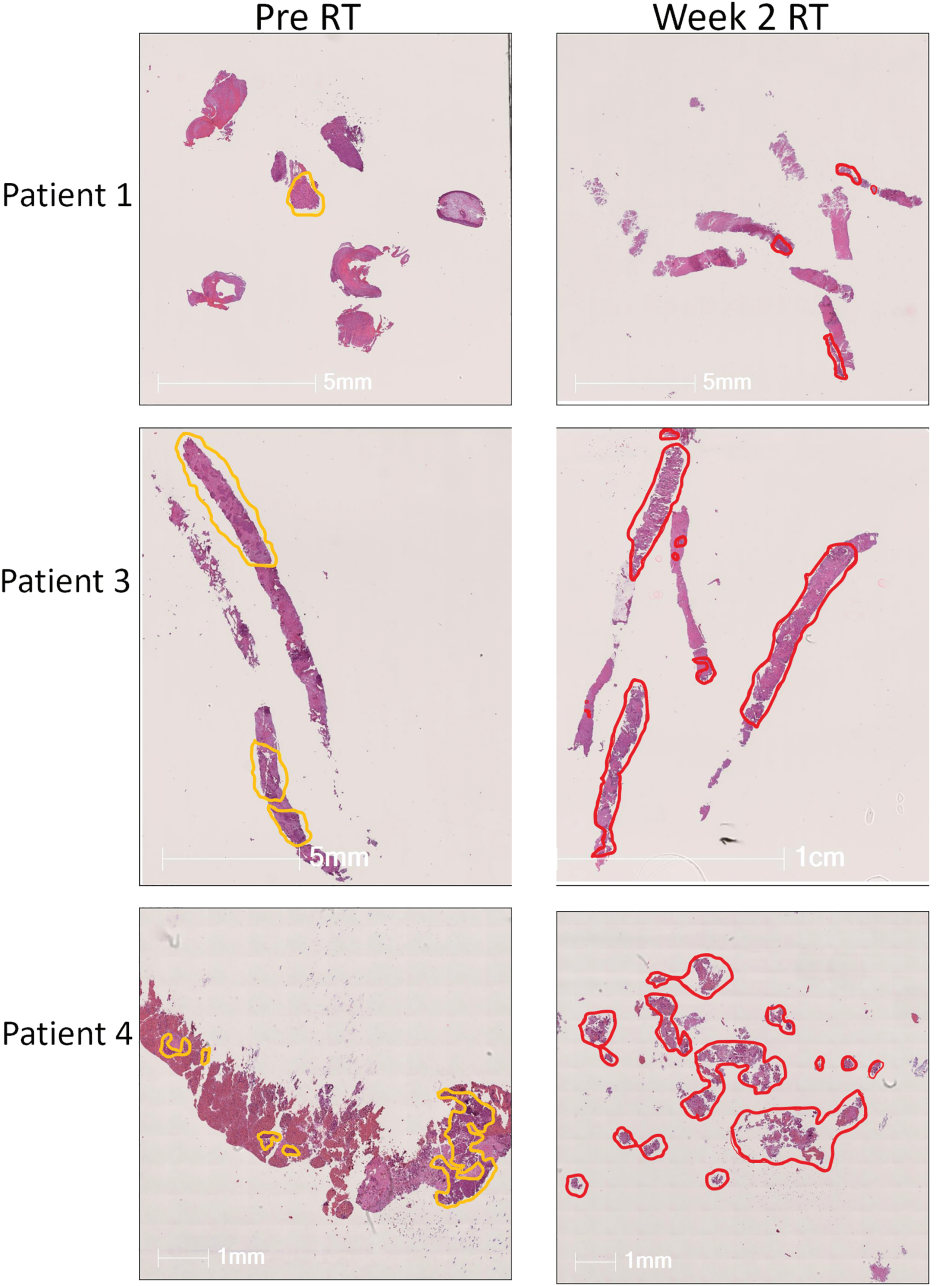
Annotation of tumor biopsies. Hematoxylin and Eosin stain (H&E) from lung tumor biopsies at baseline (left-hand column) and in week 2 of RT (right-hand column) from patients 1, 3, and 4 in the PD-RAD study. Tumor and stromal areas were marked by the pathologist and are annotated in yellow (baseline) or red (wk2 of RT). Wk, week

Tumor biopsies collected pre-RT and at Week 2 of RT were stained using the Ultivue Immuno-8 kit. Tumor and stromal regions of interest were annotated by a histopathologist, with variations between biopsies seen (Fig. 2). Patient 1 had minimal tumor tissue in both pre-RT and Week 2-RT samples. For Patient 3, tumor and stromal areas were relatively well-preserved, and Patient 4 had significant tissue fragmentation and differences in tissue architecture between pre-RT and Week 2-RT samples.

**Figure 3:**
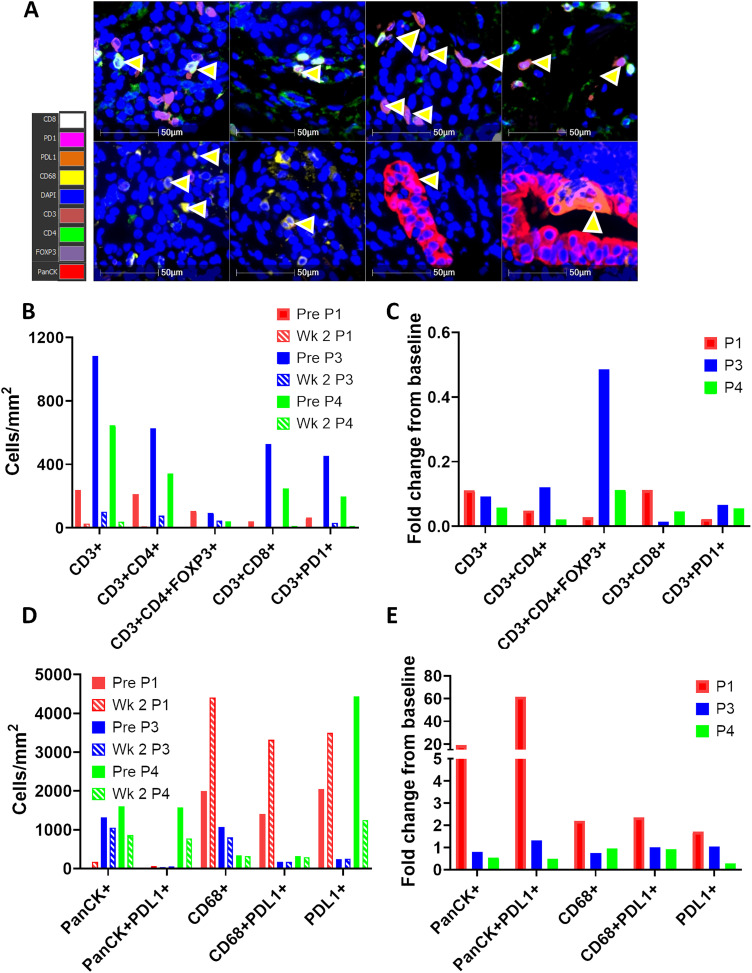
Macrophages but not T cells increase in the tumor microenvironment (TME) post radiotherapy in Patient 1. (**A**) Representative images taken in Halo software of lung biopsies from Patient 1 stained with the Ultivue Immuno-8 kits. Yellow arrows illustrate cells positive for the 8 markers (colour key for 8 markers is shown on left hand panel). Nuclei are stained with DAPI (blue), and PanCK^+^ tumor cells are visualised in red. (**B**–**E**) Lung biopsies at baseline and week 2 of RT from Patients 1, 3 & 4 were stained by multiplex IHC (Ultivue), and positivity for the 8 markers was set using Halo software. The density of cells of 10 different phenotypes was calculated and is depicted at baseline (shown as solid bars) and week 2-RT (shown as hashed bars) as cells/mm^2^ (**B**,**D**) and fold change from baseline (**C**,**E**). Data is shown for Patient 1 (P1, red bars), Patient 3 (P3, blue bars), and Patient 4 (P4, green bars). TME, tumor microenvironment

When analyzing the effects of RT on T cells within the TME (Fig. 3A), it was observed that total T cells, CD4^+^, CD8^+^, PD1^+^, and FoxP3^+^ T cell populations decreased in the TME between baseline and week 2 of RT (Fig. 3B,C) in all 3 patients. However, when quantifying macrophages and tumor cells (Fig. 3D), the opposite was seen in one of the three patients (Patient 1), where there was a 1.7-fold increase in total PD-L1^+^ cells, 61.7-fold increase in PD-L1^+^ PanCK^+^ tumor cells, 2.2-fold increase in CD68^+^ macrophages, and 2.35-fold increase in PD-L1^+^ CD68^+^ macrophages post-RT (Fig. 3E). Additionally, whilst PanCK^+^ and PanCK^+^PD-L1^+^ cell densities dramatically increase between baseline and wk-2 of RT in Patient 1 (19 and 62-fold respectively), these populations are decreased in Patients 3 and 4 after two weeks of RT. However, it is notable that, despite this increase, the overall tumor cell densities in Patient 1 remained low compared with those seen in Patients 3 and 4 (179 vs. 1055 vs. 860 cells/mm^2^ for panCK^+^, Fig. 3D), which tallies with the good clinical response Patient 1 achieved and suggests that despite an increase in tumor cell density during RT, the overall low tumor proportion might be a contributing factor to good patient outcome. Additionally, whilst a 1.7-fold increase in total PD-L1^+^ cells was seen in Patient 1, no increase was seen in Patient 3, and Patient 4 had a 0.28-fold reduction in PD-L1^+^ cells during RT. Furthermore, in comparison to Patient 1, which saw an increase, CD68^+^ macrophages decreased or remained constant in Patients 3 and 4 (Fig. 3D,E). Macrophages were also present in the TME at higher levels at baseline and Week 2 of RT for Patient 1 (2000 and 4410 cells/mm^2^) compared to Patient 3 (1000 and 687 cells/mm^2^) and Patient 4 (337 and 323 cells/mm^2^) (Fig. 3D).

Although the number of patients recruited to the study are too low for any meaningful statistical analysis, a simple linear regression was performed to see if there was any correlation between the composition of cells in the TME at baseline and Week 2-RT and progression free survival (Fig. S1) as some studies have shown that a minimum of two subjects per variable (SPV) are required to estimate coefficients with low bias [16]. Whilst no definitive conclusions can be drawn with only three data subjects per variable, a strong negative correlation was seen between the density of panCK^+^ tumor cells at baseline (R^2^ = 0.86, *p* = 0.2438) and Week 2 of RT (R^2^ = 1, *p* < 0.0001), and a strong positive correlation was seen between PD-L1^+^ cells at Week 2 of RT and PFS (R^2^ = 0.99, *p* = 0.0577), but not at baseline (Fig. S1A). Interestingly, it appeared to be PD-L1 expression on CD68^+^ macrophages rather than panCK^+^ tumor cells, which was positively correlated with PFS (Fig. S1B). When PD-L1 expression on macrophages only was analyzed, a strong positive correlation with PFS was seen both at baseline and Week 2-RT. Unexpectedly, there was a negative correlation between CD3^+^/CD3^+^CD8^+^ T cells and PFS at baseline. Whilst some markers appeared to show a good correlation between cell density and PFS, *p*-values were often not significant, which could be due to the small sample size rather than a true lack of correlation.

Whilst some potentially interesting findings were seen in the TME in biopsies obtained during RT, the requirement to undergo an additional invasive biopsy procedure during treatment significantly hindered patient recruitment to the PD-RAD study. Liquid biopsies (such as peripheral blood sampling) are far more acceptable to patients, and so we analyzed patient blood samples collected during treatment to determine if any systemic immune changes might correlate with immune changes in the TME or patient outcomes.

### 3.3 Immuno-Phenotyping of PBMC during RT by CyTOF

**Figure 4:**
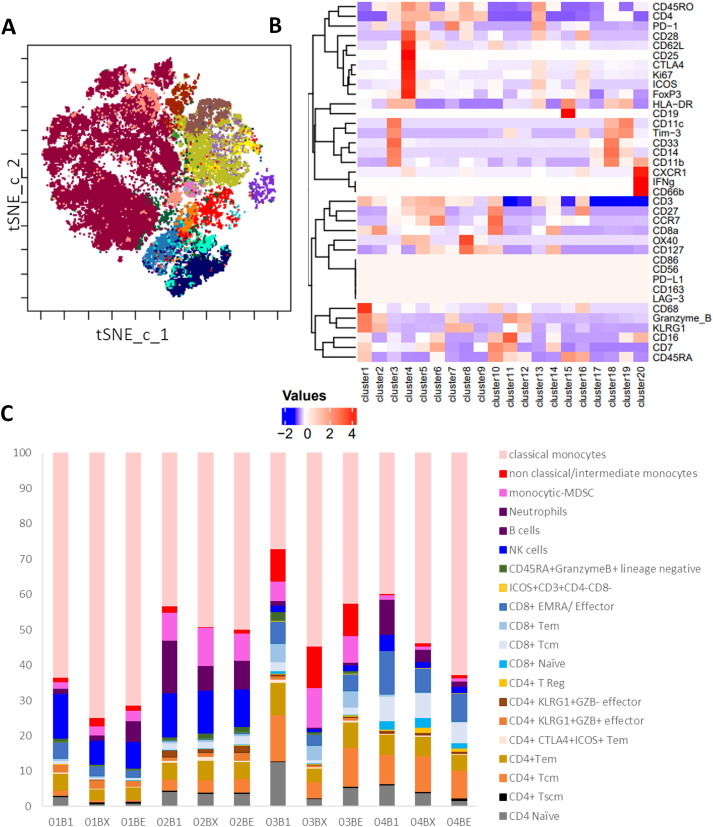
Cytof analysis of changes in PBMC populations during RT. Patient PBMC isolated at baseline (B1), Wk2 of RT (BX) and end of RT (BE) from patients 1–4 were analyzed by mass cytometry and following t-SNE-CUDA dimensionality reduction, unsupervised clustering of CD45^+^ cells using FLOWSOM was performed. (**A**) The 20 metaclusters were visualized on a tSNE-CUDA plot, and a representative example is shown for Patient 1 at baseline. (**B**) Heatmap of median expression of each marker in each metacluster across all 12 samples using a Z-score standardization method used to allocate cell phenotypes to each metacluster. (**C**) The percentage of cells in each metacluster for Patients 1–4 at baseline (B1), Wk 2 of RT (BX) and end of RT (BE)

In order to determine whether RT induced detectable systemic changes to immune cell populations, PBMC isolated from patients were examined by mass cytometry CyTOF. Fig. 4A demonstrates the 20 metaclusters identified following tSNE-CUDA dimensionality reduction and FlowSom clustering analysis. The overall median expression intensity of functional markers in each cluster from all samples was displayed in a heatmap (Fig. 4B), and this was used to define downstream immune cell phenotypes in each cluster. The expression intensities of functional markers including Tim-3, CD68, CXCR1, CD25, OX40, CD11c, IFN-γ, CD163, CD86, LAG-3, CD56, PD-L1, CD28 were low in all cell populations. Cluster identification was mainly performed using CD14, CD16, CD3, CD4, CD8, CD19, CD11b, CD33, CD62L, CCR7, CD45RO and CD45RA. Additional clusters were identified based on KLRG1, PD1, CTLA4, and ICOS expression, which were predominantly localized on CD4 or CD8 T cell subsets, whilst Granzyme B was expressed on T cells and neutrophils/myeloid-derived suppressor cells (MDSCs). CTLA4, Ki67, and ICOS were expressed only within certain CD4 T cell clusters.

The percentages of each cell cluster amongst the total cell population of each patient at baseline, during week 2-RT, and end-RT are demonstrated in Fig. 4C. Classical monocytes were the dominant cell phenotype in all samples, consistently representing the largest proportion across pre-RT, Week 2-RT, and end-RT, and furthermore, they increased during RT in all four patients. In contrast, the total population of monocytes comprises only 5%–20% of PBMC in healthy donors, suggesting that patients with NSCLC have elevated levels of monocytes in their blood. However, variations in other immune cell populations are apparent over time, although results must be interpreted with caution due to low patient numbers. At Week 2-RT, there is an increase in non-classical/intermediate monocytes in certain patient samples, particularly in Patient 3 (03BX), indicating potential shifts in innate immune responses during treatment. Additionally, some T cell subsets, such as CD8^+^ effector memory cells (CD8^+^ EMRA) and CD4^+^ regulatory T cells (CD4^+^ Tregs), show proportional increases in specific patients during week 2-RT (e.g., Patient 2 (02BX) and Patient 3 (03BX)). By the end of RT, these changes are less pronounced, with a reversion toward pre-RT proportions in several immune subsets.

**Figure 5:**
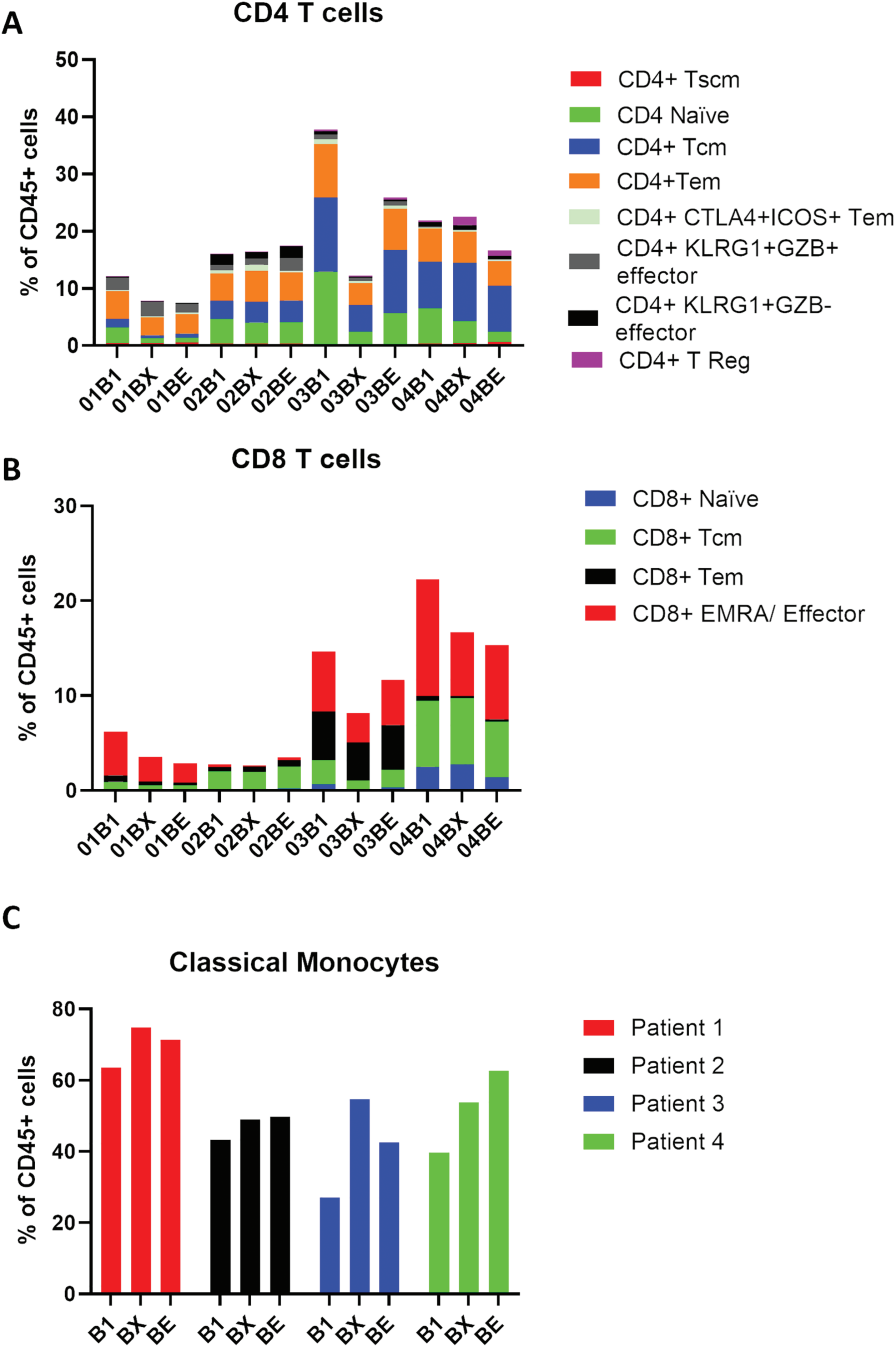
Classical monocytes increase in the blood during RT treatment of NSCLC. Patient PBMC (patients 1–4) isolated at baseline (B1), Wk2 of RT (BX), and end of RT (BE) were analyzed by mass cytometry, and following tSNE-CUDA dimensionality reduction, unsupervised clustering of CD45^+^ cells using FLOWSOM was performed. (**A**) The percentage of PBMC in clusters designated CD4^+^ is shown for each patient. (**B**) The percentage of PBMC in clusters designated as CD8^+^ is shown for each patient. (**C**) The percentage of PBMC in a putative classical monocyte cluster (CD14^+^CD16^−^, HLA-DR^+^) is shown for each patient. B1 = baseline, BX= Week 2-RT, BE = end RT. Patient 1 (red bars), Patient 2 (black bars), Patient 3 (blue bars), Patient 4 (green bars). HLA, Human Leukocyte Antigen-DR

In order to show the trends in immune cell dynamics more clearly, the proportions of CD4^+^ T cells, CD8^+^ T cells, and classical monocytes were separated into distinct bar plots (Fig. 5). Notably, Patient 1 displayed the lowest levels of CD4 and CD8 T cells pre-treatment amongst the cohort. Regulatory T cells (CD4^+^T Regs) remain very low in all patients throughout RT, with proportions consistently below 1% of total CD45^+^ cells, except in Patient 4. Interestingly, Patient 4 showed a clear increase in T Regs during RT, peaking at approximately 2%–3% by Week 2-RT before stabilizing at the end of RT (Fig. 5A). Central memory T cells (CD4^+^Tcm) exhibited relatively higher proportions in Patients 3 and 4 compared to Patient 1, particularly at pre-RT, where Tcm constituted approximately 15%–20% of total CD45^+^ cells. However, during RT, a decreasing trend in Tcm was observed in these patients. Effector memory CD4^+^ T cells (Tem) remained stable in most patients but declined during RT in Patient 3, followed by a recovery at the end of RT. Interestingly, the proportion of CD8^+^ EMRA T cells decreased in all patients during RT, including Patient 1, who had the best clinical outcome (Fig. 5B). However, Patients 3 and 4, classified as non-responders, exhibited consistently higher proportions of CD8^+^ EMRA T cells compared to Patient 1 at all time points, and Patient 3 (who had the worst clinical outcome) also had much higher numbers of CD8^+^ Tem at all time points than the other patients. Classical monocytes increased in all patients during Week 2-RT, but the baseline levels and magnitude of change vary (Fig. 5C). Patient 1 consistently had the highest proportions, rising from 60% pre-RT to 80% at Week 2-RT, then stabilizing at 70% by end-RT. In Patient 3, the baseline was lower (~30% pre-RT), but the relative increase was greater, peaking at 65% during Week 2-RT, though still below the levels observed in Patient 1.

In order to understand the prognostic value of specific immune cell subsets, the correlations between their proportions in PBMCs at different time points (baseline, Week 2-RT, and end-RT) and PFS were analyzed using linear regression (Fig. S2), albeit with the caveat that the statistical power is weak due to low patient numbers. At baseline, a strong negative correlation was observed between the percentage of CD4^+^ T cells and PFS (R^2^ = 0.8, *p* = 0.1003), suggesting that higher baseline levels of CD4^+^ T cells are associated with shorter PFS. This trend was less pronounced at Week 2-RT (R^2^ = 0.14, *p* = 0.6215) but re-emerged with moderate strength at end-RT (R^2^ = 0.6, *p* = 0.2233). For CD8^+^ T cells, as with CD8^+^ cells in the TME, a strong negative correlation at baseline (R^2^ = 0.7, *p* = 0.1651) was seen, indicating that higher initial levels were associated with shorter PFS. The correlation weakened slightly at Week 2-RT (R^2^ = 0.52, *p* = 0.2768) but strengthened again by end-RT (R^2^ = 0.82, *p* = 0.0895), suggesting that CD8^+^ T cell levels at later stages of RT may have predictive value for disease progression. Classical monocytes showed a positive correlation with PFS at baseline (R^2^ = 0.7, *p* = 0.1624), indicating that higher baseline levels are associated with longer PFS. This correlation diminished during treatment, with weaker positive associations observed at Week 2-RT (R^2^ = 0.17, *p* = 0.5807) and end-RT (R^2^ = 0.246, *p* = 0.5031).

## 4 Discussion

The PD-RAD study was a prospective study aiming to investigate immunomodulatory changes in both peripheral blood mononuclear cells (PBMCs) and the TME in NSCLC patients undergoing radiotherapy (RT), which was prematurely terminated secondary to the COVID-19 pandemic, having recruited only 6 patients. Whilst the study did not meet its primary endpoint of 21 out of 30 evaluable participants, it demonstrated some feasibility of collecting tumor and blood samples in this clinical setting. Exploratory endpoints included assessing changes in PD-L1 expression and monitoring immune dynamics in both the TME and PBMCs. Whilst limited by the small sample size, the study produced several hypothesis-generating findings which are worthy of further exploration in larger patient cohorts.

The mIHC analysis of NSCLC tumors revealed an increase in PD-L1 expression between the pre- and during RT biopsies in both tumor cells and macrophages in a patient who responded well to treatment (Patient 1). In contrast, no increase in PD-L1 expression was observed in two patients who experienced progressive disease. Although confirmation is needed in larger numbers of patients, it is possible that PD-L1 is not consistently upregulated across all patients in the TME following 2 weeks of CRT, potentially having implications for the scheduling of ICI and CRT in NSCLC patients and explaining conflicting results in clinical trials. Whilst the number of patients in the study is too low for accurate statistical analysis, preliminary linear regression suggests that the density of PD-L1^+^ macrophages (CD68^+^ cells) might correlate with PFS, while PD-L1 expression on tumor cells appeared less relevant. This suggests that macrophage-associated PD-L1 expression may play a more critical role than tumor cell PD-L1 expression in driving treatment outcomes. Elevated levels of PD-L1^+^CD68^+^ macrophages have been shown to be predictive for OS of NSCLC patients receiving anti-PD-1 treatment, supporting this notion [17]. Furthermore, this correlation was seen both at baseline and Wk-2 of RT, suggesting that further studies interrogating the potential role of PD-L1^+^ CD68^+^ macrophages in response to CRT could be performed on diagnostic biopsies, alleviating the requirement to collect challenging on-treatment lung biopsies. These findings align with the hypothesis that PD-L1 induction within the TME may enhance immune-mediated tumor control in some patients. Supporting the lack of correlation between tumor cell PD-L1 expression and PFS, reports in the literature suggest that tumor cell PD-L1 expression might even be detrimental. For example, increased PD-L1 expression in circulating tumor cells during RT has been shown to correlate with worse prognosis in NSCLC [18], and lower baseline tumor PD-L1 expression has also been associated with better prognosis in locally advanced NSCLC patients receiving CCRT [12]. In contrast, another study identified tumoral PD-L1 expression to be increased in the majority of NSCLC patients after CRT, but no correlation with survival could be found [11]. Preclinical studies have shown that RT can upregulate PD-L1 expression on tumor cells, with acquired resistance to fractionated RT being overcome by concurrent PD-L1 blockade mediated by interferon-γ-producing CD8^+^ T cells [10,19]. We know that PD-L1 has an established role as a predictive biomarker for response to ICIs (11), where patients with high PD-L1 expression are more likely to achieve better PFS and OS. Thus, the phenomenon is recognized as the ‘PD-L1 paradox’—where high PD-L1 expression is associated with worse prognosis in early NSCLC [9] and perhaps less so in advanced NSCLC [20,21] but equally represents an attractive treatment target. Therefore, PD-L1 as a biomarker remains challenging due to variability in quantification platforms, heterogeneity between diagnostic and surgical biopsies, and the dynamic nature of PD-L1 expression, which can be influenced by tumor-immune interactions and treatments such as chemotherapy, targeted therapy, or RT. Additionally, differing results highlight that PD-L1’s prognostic and predictive values are rather more dynamic than static, and highly dependent on the specific clinical scenario and the concurrent therapeutic landscape.

Further analysis revealed that tumor cell density (Pan-CK^+^ cells) negatively correlated with PFS, particularly during RT, highlighting its potential as a simple prognostic biomarker. Whilst this was highly significant at Wk-2 of RT (r^2^ = 1), it must be stressed that with only 3 patients, definitive conclusions regarding correlations with PFS cannot be drawn, as linear regression is commonly thought to require at least 10–15 subjects per variable for accurately estimating correlation. Additionally, the density of T cells (CD3^+^ cells) within the TME also negatively correlated with PFS, with T cell levels decreasing during RT. This finding suggests that myeloid cells, specifically macrophages, may have a more pivotal role than T cells in mediating the therapeutic response to RT in this context. Future studies would benefit from investigating other myeloid populations, such as neutrophils and MDSCs, to further elucidate their contributions.

High-dimensional mass cytometry (CyTOF) analysis of blood samples provided complementary insights using a less invasive liquid biopsy approach. A notable increase in CD14^+^CD16-HLA-DR^+^ classical monocytes was observed during RT, with the highest levels detected in the patient with the best response. This suggests that classical monocytes may serve as a predictive biomarker for RT response, consistent with prior studies linking pre-treatment levels of classical monocytes to improved outcomes with immune checkpoint inhibitors [22,23]. This increase in classical monocytes occurred without a corresponding rise in T cells within the TME or blood, suggesting a potential T cell-independent mechanism of action in the absence of ICI therapy. We found that T-cell populations are generally present in the blood during radiotherapy, which is in concordance with other studies, which demonstrate that radiotherapy, but not chemotherapy, leads to lower levels of systemic lymphocytes [24].

The CyTOF platform also identified multiple immune cell subsets, including classical monocytes, non-classical/intermediate monocytes, and a hybrid classical monocyte/dendritic cell subset, demonstrating its capability to resolve immune heterogeneity. These findings align with prior studies associating classical monocytes, NK cells, and ICOS^+^ CD4 T cells with treatment efficacy in NSCLC [23,25]. The ability of CyTOF to identify rare and diverse immune subsets underscores its value as a robust tool for immune profiling in a less invasive, reproducible, and cost-effective manner. The careful selection of immune marker panels will be critical to maximizing their efficacy in monitoring immune dynamics.

The limitations of this study stem primarily from a small sample size, challenges in sample availability, and clinical variability. Small sample size precluded definitive statistical conclusions. Linear regression was selected as an exploratory tool to assess potential associations between continuous variables, but the resulting models are inherently underpowered and prone to overfitting. Consequently, any apparent relationships should be viewed as hypothesis-generating and validated in larger, prospective studies. Invasive biopsies, particularly of the lung and in patients who may have respiratory compromise or other comorbidities, prove an ongoing challenge, and even when a biopsy is possible, variability in the quantity of tumor material obtained can complicate inter-patient comparisons. For example, Patient 1 had limited biopsy material with few areas of viable tumor, but achieved the best outcome. This raises questions about whether biopsies with less tumor material inherently reflect less aggressive disease and lower disease burden or whether biopsy material size is random and offers an incomplete picture of the TME. It is essential to acknowledge that variability in tumor material is a common obstacle in the development of predictive and prognostic biomarkers requiring tissue collection. For example, the biopsies collected from Patient 4 were obtained from fine needle aspirates from lymph node metastases rather than core needle biopsies, and so it is unknown how the TME might differ between these two sample types and tumor sites. Some studies employ tissue microarrays (TMAs), which utilize smaller tissue samples than those analyzed here. Overall, further investigation is necessary to validate these results. Nonetheless, this study has informed a larger prospective study named TIMM-RAD, which is currently recruiting samples from patients with five different tumor types undergoing RT (ClinicalTrials.gov Identifier: NCT05076500) and aims to collect 50 to 100 patients per arm to generate statistically robust data, aiming to identify immune biomarkers of RT response. The advancement in technologies such as spatial transcriptomics and imaging mass cytometry now means that a wealth of information can be obtained from biopsies taken during/post RT [7,26]. The development of robotic bronchoscopies offers the potential for safer, higher-quality lung cancer biopsies, which would improve the feasibility of the study today [27,28]. In future work, gene profiling could be explored if there is enough tissue, considering the potential application of tumor heterogeneity and gene profiling for developing biomarkers in lung cancer [29,30].

## 5 Conclusions

In conclusion, this study profiles a range of immune cell populations in NSCLC patients at serial timepoints during a course of curative-intent RT over 4 weeks. The findings suggest the potential role of macrophages, particularly PD-L1^+^ macrophages, and the CD14^+^CD16-HLA-DR^+^ classical monocytes in driving treatment responses, as well as the correlation between classical monocytes in the blood and therapeutic response. Due to the small number of patients, these findings should not be over-interpreted and are at best hypothesis-generating. While limited by the small sample size, this study provides valuable insights into immune-mediated responses to radiotherapy and identifies potential biomarkers for further investigation, particularly the potential biomarkers identified in diagnostic biopsies and blood samples. These observations warrant further investigation in larger cohorts to validate the findings and explore their potential utility as immune biomarkers of response to RT.

## Supplementary Materials



## Data Availability

Data not available due to ethical restrictions. “Due to the nature of this research, participants of this study did not agree for their data to be shared publicly, so supporting data is not available”.

## Supplementary Materials

The supplementary material is available online at https://www.techscience.com/doi/10.32604/journal.2025.0xxxxx/s1.
