# Supplementary material for: Investigating the Immune Effects of Radiotherapy in Non-Small Cell Lung Cancer—Results of the PD-RAD Study
Source: Oncol Res. 2026 Feb 24;34(3):14. doi: 10.32604/or.2025.072053 (PMC12963685; doi:10.32604/or.2025.072053)
Supplement: Supplementary file 1 [file OncolRes-34-72053-s001.docx]

**Table S1** CyTOF immune profiling panel: list of metal-tagged antibodies, including targets, clones, catalog numbers, manufacturer information and dilution ratios.

| **Metal** | **Target** | **Clone** | **Cat. Number** | **City, Province/State, Country** | **dilution ratios** |
| --- | --- | --- | --- | --- | --- |
| 89Y | CD3 | UCHT1 | in house^∆^ | CRUK MI, Manchester, UK | 1 in 50 |
| 113In | CD11c | 3.9 | in house^∆^ | CRUK MI, Manchester, UK | 1 in 50 |
| 115In | CD163 | GHI/61 | in house^∆^ | CRUK MI, Manchester, UK | 1 in 50 |
| 141Pr | CD45 | HI30 | 3141009B | Cambridge, UK | 1 in 50 |
| 142Nd | CXCR1 |  | 3142009B | Cambridge, UK | 1 in 50 |
| 143Nd | CD127 | A019D5 | 3143012B | Cambridge, UK | 1 in 50 |
| 144Nd | CD11b | ICRF44 | 3144001B | Cambridge, UK | 1 in 200 |
| 145Nd | CD4 | RPA-T4 | 3145001B | Cambridge, UK | 1 in 100 |
| 146Nd | CD8 | RPA-T8 | 3146001B | Cambridge, UK | 1 in 50 |
| 147Sm | CD7 | CD7-6B7 | 3147006B | Cambridge, UK | 1 in 50 |
| 148Nd | CD16 | 3G8 | 3148004B | Cambridge, UK | 1 in 100 |
| 149Sm | CD25 | 2A3 | 3149010B | Cambridge, UK | 1 in 50 |
| 150Nd | LAG3 | 1C3C65 | 3150030B | Cambridge, UK | 1 in 50 |
| 151Eu | CD14 | M5E2 | 3151009B | Cambridge, UK | 1 in 50 |
| 152Sm | CD66b | 80H3 | 3152011B | Cambridge, UK | 1 in 50 |
| 153Eu | CD62L | DREG-56 | 3153004B | Cambridge, UK | 1 in 50 |
| 154Sm | Tim3 | F38-2E2 | 3154010B | Cambridge, UK | 1 in 50 |
| 155Gd | CD45RA | HI100 | 3155011B | Cambridge, UK | 1 in 50 |
| 156Gd | PD-L1 | 29E.2A3 | 3156026B | Cambridge, UK | 1 in 50 |
| 158Gd | OX40 | ACT35 | 3158012B | Cambridge, UK | 1 in 50 |
| 159Tb | CCR7 | G043H7 | 3159003A | Cambridge, UK | 1 in 50 |
| 160Gd | CD28 | CD28.2 | 3160003B | Cambridge, UK | 1 in 100 |
| 161Dy | CTLA-4* | 14D3 | 3161004B | Cambridge, UK | 1 in 50 |
| 162Dy | FoxP3* | PCH101 | 3162024A | Cambridge, UK | 1 in 50 |
| 163Dy | CD33 | WM53 | 3163023B | Cambridge, UK | 1 in 50 |
| 164Dy | CD45RO | UCHL1 | 3164007B | Cambridge, UK | 1 in 100 |
| 165Ho | IFNγ*° | B27 | 3165002B | Cambridge, UK | 1 in 50 |
| 166Er | KLRG1 | 2388C | in house^∆^ | CRUK MI, Manchester, UK | 1 in 50 |
| 167Er | CD27 | L128 | 3167006B | Cambridge, UK | 1 in 50 |
| 168Er | ICOS | C398.4A | 3168024B | Cambridge, UK | 1 in 50 |
| 169Tm | CD19 | HIB19 | 3169011B | Cambridge, UK | 1 in 50 |
| 170Er | CD3e | UCHT1 | 3170001B | Cambridge, UK | 1 in 50 |
| 171Yb | CD68° | Y1/82A | 3171011B | Cambridge, UK | 1 in 50 |
| 172Yb | Ki67* | B56 | 3171011B | Cambridge, UK | 1 in 50 |
| 173Yb | Granzyme B* | GB11 | 3173006B | Cambridge, UK | 1 in 50 |
| 174Yb | HLA-DR | L243 | 3174001B | Cambridge, UK | 1 in 50 |
| 175Lu | PD1 | EH12.2H7 | 3175008B | Cambridge, UK | 1 in 50 |
| 176Yb | CD56 | NCMA16.2 | in house^∆^ | CRUK MI, Manchester, UK | 1 in 50 |

All antibodies were mouse anti-human and were purchased from Fluidigm (Standard BioTools).

*Intracellular markers: IFNγ, CD68, Ki67, CTLA4, FoxP3, granzyme B

°Surface and intracellular markers: IFNγ, CD68.

^∆^ in house, manually conjugated antibody; CRUK Manchester Institute core facilities.


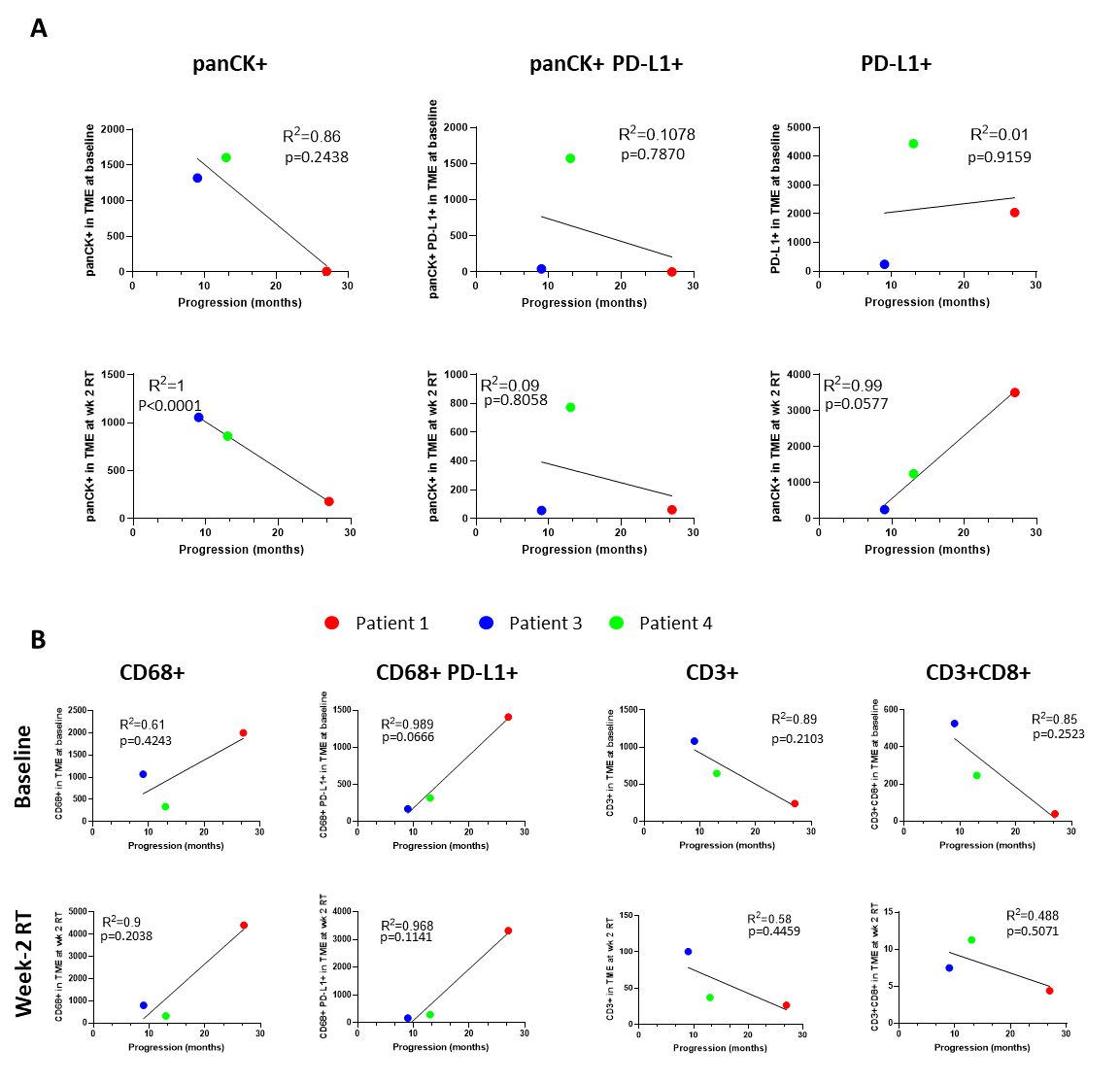


**Figure S1.** **The density of tumor cells and PD-L1+ cells in the TME appears to correlate with progression free survival (PFS).** Lung biopsies at baseline and week 2 of RT from Patients 1, 3 & 4 were stained by multiplex IHC (Ultivue) and positivity for the 8 markers was set using Halo software. The density of cells of 10 different phenotypes was calculated. Linear regression analysis of the density of various cell phenotypes at baseline and week 2 of RT compared to the patient’s PFS (months) was performed. R^2^ is shown for each regression analysis and the p value is shown when the slope is significantly non-zero (p<0.05) or close to significant (p<0.1). **(A)** Linear regression analysis for PanCK+ tumor cells, PD-L1+ PanCK+ tumor cells and total PD-L1+ cells. **(B)** Linear regression analysis for macrophages (CD68+ and CD68+PD-L1+) and total T cells (CD3+) and CD8+ T cells. PD-L1, Programmed Death-Ligand 1, TME, Tumor Microenvironment, RT, Radiotherapy, IHC, immunohistochemistry.


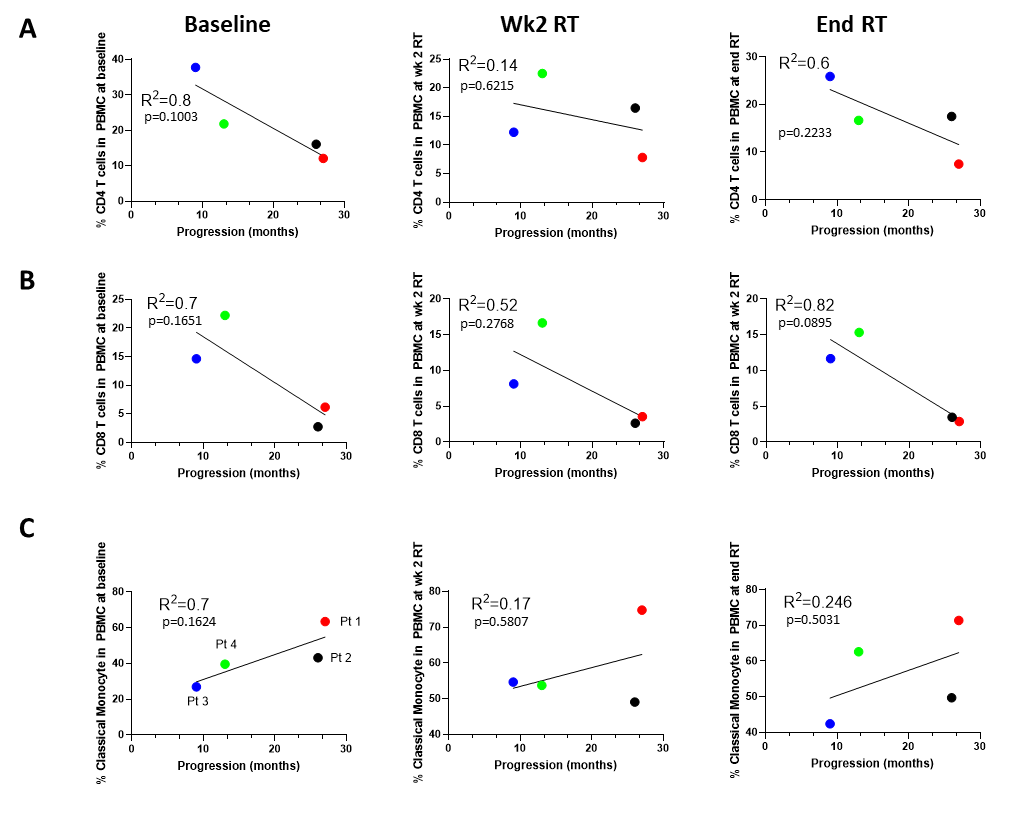


**Figure S2. The level of T cells and classical monocytes in the peripheral blood shows a moderate negative and positive correlation with PFS.** Patient PBMC isolated at baseline (B1), week 2 of RT (BX) and end of RT (BE) from patients 1-4 were analyzed by mass cytometry and following tSNE-CUDA dimensionality reduction, unsupervised clustering of CD45+ cells using FLOWSOM was performed. Cell phenotypes were identified by median expression of markers within each cluster and the percentage of PBMC in each cluster/phenotype was calculated for patients 1-4 at baseline, week 2 of RT and end of RT. Linear regression analysis of the percentage of PBMCs which are CD4^+^ **(A)**, CD8^+^ **(B)** or classical monocytes **(C)** at baseline and week 2 of RT compared to the patients PFS (months) was performed. R^2^ is shown for each regression analysis, and the p value is shown when the slope is significantly non-zero (p<0.05) or close to significant (p<0.1). **(A)** CD4+ T cell clusters, **(B)** CD8+ T cell clusters, **(C)** classical monocyte cluster (CD14+CD16-). PBMC, peripheral blood mononuclear cells.
